# Anti-Cancer Activity of Phytochemicals Targeting Hypoxia-Inducible Factor-1 Alpha

**DOI:** 10.3390/ijms22189819

**Published:** 2021-09-10

**Authors:** Ba Da Yun, Seung Wan Son, Soo Young Choi, Hyo Jeong Kuh, Tae-Jin Oh, Jong Kook Park

**Affiliations:** 1 Department of Biomedical Science and Research Institute for Bioscience & Biotechnology, Hallym University, Chunchon 24252, Korea; dbsbada@naver.com (B.D.Y.); miyanae@naver.com (S.W.S.); sychoi@hallym.ac.kr (S.Y.C.); 2 Department of Medical Life Sciences, College of Medicine, The Catholic University of Korea, Seoul 06591, Korea; hkuh@catholic.ac.kr; 3 Department of Pharmaceutical Engineering and Biotechnology, SunMoon University, 70 Sunmoon-ro 221, Tangjeong-myeon, Asan-si 31460, Korea; tjoh3782@sunmoon.ac.kr

**Keywords:** naturally derived compounds, phytochemical, hypoxia, normoxia, HIF, cancer

## Abstract

Hypoxia-inducible factor-1 alpha (HIF-1α) is overexpressed in cancer, leading to a poor prognosis in patients. Diverse cellular factors are able to regulate HIF-1α expression in hypoxia and even in non-hypoxic conditions, affecting its progression and malignant characteristics by regulating the expression of the HIF-1α target genes that are involved in cell survival, angiogenesis, metabolism, therapeutic resistance, et cetera. Numerous studies have exhibited the anti-cancer effect of HIF-1α inhibition itself and the augmentation of anti-cancer treatment efficacy by interfering with HIF-1α-mediated signaling. The anti-cancer effect of plant-derived phytochemicals has been evaluated, and they have been found to possess significant therapeutic potentials against numerous cancer types. A better understanding of phytochemicals is indispensable for establishing advanced strategies for cancer therapy. This article reviews the anti-cancer effect of phytochemicals in connection with HIF-1α regulation.

## 1. Introduction

Hypoxia-inducible factors (HIFs) are transcription factors that contain α and β subunits. HIF-1α and HIF-1β subunits form HIF-1 heterodimers. In addition, HIF-2 complexes are made up of HIF-2α and HIF-1β subunits [1]. A beta subunit shows a constitutively expressed pattern, whereas the expression of alpha subunits is oxygen-dependently regulated. In normoxic conditions, HIF-1α and HIF-2α can be hydroxylated by prolyl hydroxylases (PHDs). Hydroxylated α subunits are ubiquitinated by von Hippel Lindau E3 ubiquitin ligase (*VHL*) and consequently degraded by proteasomes [2]. Under hypoxic conditions, PHDs are inactivated due to the deficiency of oxygen. Therefore, the transcriptional activities of HIFs are promoted [2].

In addition to this, the level of α subunits can be modulated by several different factors. For instance, phosphatase and tensin homolog (*PTEN*) is able to promote HIF-1α degradation by mouse double min 2 homolog (*MDM2*) in hypoxic conditions. Accumulation of HIF-1α is interrupted by the inhibition of phosphoinositide 3-kinase (PI3K)/AKT [3]. Besides, PI3K/AKT/mechanistic target of rapamycin (mTOR) signaling activates HIF-1α expression in both normoxic and hypoxic conditions. Specifically, the translation of HIF-1α messenger RNA (mRNA) is enhanced by the PI3K/AKT/mTOR pathway [4]. Moreover, HIF-1α can be upregulated via nitric oxide and signal transducer and activator of transcription 1 (*STAT1*) in response to doxorubicin under normoxia [5]. In the case of HIF-2α, it was demonstrated that OTU deubiquitinase 7B (*OTUD7B*) can positively regulate the transcription of HIF-2α by stabilizing an E2F transcription factor 1 (*E2F1*) independently of hypoxia [6].

HIFs exert influence on diverse cellular events by transcriptionally controlling a broad range of genes [7,8]. HIF-1α and HIF-2α suppress apoptotic cell death induced by the tumor protein p53 (*TP53*) [9,10]. HIFs can promote stemness and epithelial-to-mesenchymal transition (EMT), thus supporting cancer aggressiveness and metastasis [11,12]. HIFs also contribute to angiogenesis and energy metabolism via transcriptionally regulating target genes, such as vascular endothelial growth factor (VEGF) and glucose transporter 1 (*GLUT1*) [13,14,15]. Other cellular events controlled by HIFs include therapeutic resistance and anti-cancer immunity. For example, HIFs aggravate resistance to paclitaxel and gemcitabine via inducing the expression of interleukin 6 (IL-6), IL-8, and ATP-binding cassette subfamily B member 1 (*ABCB1*, also known as multidrug resistance protein 1 (*MDR1*)) [16]. HIF-1α and HIF-2α can lead to the induction of programmed death-ligand 1 (PD-L1) and the infiltration of tumor-associated macrophages (TAMs), respectively, thereby limiting anti-cancer immunity [17,18].

It has been noticed that the expression of HIF-1a and HIF-2a is stronger in cancer than in normal tissues [19,20,21]. Therefore, targeting HIFs has been suggested as an effective strategy for cancer therapy. The silencing of HIF-1α can compensatorily upmodulate HIF-2α and vice versa [22,23]. In addition, HIF-1α and HIF-2α have target gene divergency, so they can differently regulate the transcription of downstream targets [24,25,26]. Therefore, it is desirable to dually inhibit HIF-1α and HIF-2α. Phytochemicals and their derivatives have exhibited potent anti-cancer activities by restraining progression and malignancy, such as cell proliferation and metastasis [27,28,29]. Advanced knowledge on the functional aspects of phytochemicals is invaluable to establishing better therapeutic options against cancer. This review article presents the effects of compounds derived from plants on HIF-1α in conjunction with their efficacy in cancer. The structure, source, and clinical trial status of phytochemicals stated in this review are summarized in supplementary Table S1.

## 2. Alkaloid and Organosulfur Compounds from Natural Sources

### 2.1. Alkaloids

#### 2.1.1. Berberine

**Figure 1:**
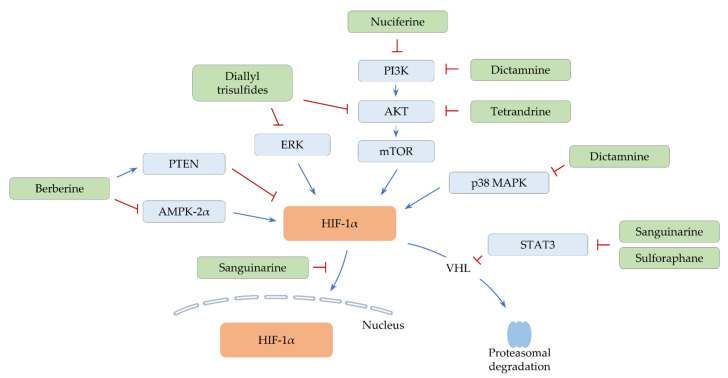
Proposed signaling pathways of HIF-1α regulation by alkaloids and organosulfurs. Positive regulation is indicated by arrow lines (blue). A negative effect is shown by perpendicular lines (red).

A clinical trial found that berberine effectively reduces the recurrence of colorectal cancer after polypectomy [30] (clinical Study Identifier: NCT02226185, Table S1). Moreover, preclinical studies have shown that berberine reverses therapeutic resistance in multiple cancers. Berberine can downmodulate the level of microRNA-93 (miR-93) that directly targets PTEN, thus sensitizing drug-resistant ovarian cancer cells to cisplatin [31]. In addition, treatment with berberine downregulates the expression of ABC transporters, including ABCB1 and ABCC1 (also known as multidrug resistance protein 1 (MRP1)), thereby improving anti-growth effects of doxorubicin on breast cancer in vivo [32]. Recently, it was reported that the level of both HIF-1α and AMP-activated protein kinase (AMPK) is reduced by berberine, alleviating hypoxia-mediated doxorubicin resistance. Co-treatment with berberine and doxorubicin significantly retards the growth of breast cancer in vivo [33]. Berberine may destabilize HIF-1α by increasing PTEN levels. In addition, AMPK is known to potentiate doxorubicin resistance in breast cancer [34], and HIF-1α can be stabilized by AMPKα2, one of the catalytic subunits of AMPK [35]. Therefore, berberine may sensitize cancer cells to doxorubicin by regulating the AMPKα2-HIF-1α axis (Figure 1 and Table 1).

**Table 1:** The list of alkaloid and organosulfur compounds that suppress HIF-1α in cancer (alphabetical order).

Compound	Compound Class	Type of Cancer	In Vitro Testing (Effective Concentrations, Cell Line, Culture Condition (Normoxia/Hypoxia))	In Vivo Experiment Model (Dose and Administration Route)	Ref.
Alkaloids					
Berberine	Benzylisoquinoline	Breast cancer	10–160 μM (human MCF-7 cell line), hypoxia (1% O_2_)	Subcutaneous injection of MCF-7 cells (5–200 mg/kg, oral)	[33]
Cyclopamine tartrate	Derivative of cyclopamine (veratrum alkaloid)	Lung cancer	15–25 μM (human H1299 cell line), normoxia	Subcutaneous injection of H1299 cells (7.5 mg/kg, intravenous)	[36]
Dictamnine	Furanoquinoline alkaloid	Colorectal cancer	10–100 μM (human HCT-116 cell line), hypoxia (1% O_2_)	Subcutaneous injection of HCT-116 cells (50–100 mg/kg, oral)	[37]
Nuciferine	Aporphine alkaloid	Colorectal cancer, Lung cancer	4–48 μM (drug-sensitive or -resistant human HCT-8 and A549 cell lines), normoxia	Subcutaneous injection of drug-resistant A549 cells (7.5 mg/kg, intraperitoneal)	[38]
Sanguinarine	Benzophenanthridine alkaloid	Breast cancer	2–4 μM (human MDA-MB-231 cell line), hypoxia (1% O_2_, cobalt (II) chloride (CoCl_2_))	-	[39]
		Breast cancer	0.5–1 μM (human T47D and MDA-MB-231 cell lines), hypoxia (1% O_2_, CoCl_2_)	Subcutaneous injection of MDA-MB-231 cells (1.25–5 mg/kg, oral)	[40]
		Hepatocellular carcinoma	0.5–2 μM (human HepG2 and SMMC-7721 cell lines), hypoxia (1% O_2_, CoCl_2_)	Subcutaneous injection of HepG2 and SMMC-7721 cells (1.25–5 mg/kg, oral)	[41]
Tetrandrine	Benzylisoquinoline	Lung cancer	5–10 μM (human A549 cell line), normoxia	-	[42]
Organosulfurs					
Diallyl trisulfide	Organic trisulfide	Breast cancer	2.5–10 μM (human MDA-MB-231 cell line), hypoxia (1% O_2_)	Microinjection of MDA-MB-231 cells into perivitelline space of zebrafish embryos (2.5–10 μM), Tail vein or orthotopic injections of MDA-MB-231 cells (25–50 mg/kg, oral)	[43]
Sulforaphane	Isothiocyanate	Bladder cancer	5–20 μM (human RT112 and RT4 cell lines), hypoxia (2% O_2_)	-	[44]
		Hepatocellular carcinoma	5–20 μM (human HepG2 cell line), hypoxia (CoCl_2_)	HepG2-bearing chick chorioallantoic membrane (CAM) assay (20 μM)	[45]

#### 2.1.2. Dictamnine

Dictamnine was reported to suppress the activity of mTOR and various mitogen-activated protein kinases (MAPKs), such as p38 MAPK and *c*-Jun *N*-terminal kinase (JNK). Moreover, dictamnine downregulates HIF-1α levels under hypoxia in vitro [37]. Treatment with dictamnine restrains cell proliferation, survival, and EMT in vitro. In addition, dictamnine hinders colorectal cancer growth in conjunction with the downregulation of HIF-1α and snail family transcriptional repressor 2 (*SNAI2*) expression in vivo [37]. Since p38 MAPK can stabilize and upregulate HIF-1α proteins [46,47], both p38 MAPK and PI3K/AKT/mTOR signaling may contribute to the regulation of HIF-1α in dictamnine-treated cells (Figure 1 and Table 1). Another study showed that dictamnine induces cell cycle arrest and that apoptotic cell death is elevated by dictamnine in combination with dihydroartemisinin [48]. These results suggest that dictamnine has multiple anti-cancer effects and that dictamnine-based combinatorial therapy may be useful to overcome hypoxia.

#### 2.1.3. Nuciferine

Several studies denoted that nuciferine inhibits diverse cellular events, such as proliferation, invasion, and EMT via suppressing the PI3K/AKT and Wnt/β-catenin signaling pathways [49,50,51]. Another study noted that nuciferine treatment results in the suppression of PI3K/AKT signaling, which in turn downmodulates the level of NF-E2-related factor 2 (*NRF2*) and HIF-1α under normoxic conditions [38] (Figure 1 and Table 1). Both NRF2 and HIF-1α can confer drug resistance via upregulating ABCB1 and ABCG2 (also known as breast cancer resistance protein, BCRP) [52,53,54,55]. Indeed, nuciferine abates the expression of ABCB1 and ABCG2, improving the anti-cancer activity of paclitaxel in vitro and in vivo [38]. Further investigation is required to investigate the effect of nuciferine on the expression of other ABC transporters and the efficacy of other anti-cancer treatments.

#### 2.1.4. Sanguinarine

STAT3 is activated by hypoxia and stabilizes HIF-1α by blocking HIF-1α binding to VHL. Therefore, HIF-1α target genes are cooperatively regulated by STAT3 [56,57]. Sanguinarine was found to disrupt the interaction between HIF-1α and STAT3 in hypoxic conditions [39]. Similarly, sanguinarine promotes proteasomal degradation of HIF-1α via inactivating STAT3 under hypoxia and impedes the growth of breast cancer in vivo [40]. In addition, sanguinarine suppresses the nuclear translocation of HIF-1α under hypoxia and restrains the HIF-1α/transforming growth factor-beta (TGF-β) feedback loop. Thus, sanguinarine significantly retards the growth and EMT progress of hepatocellular carcinoma in vivo [41]. Since TGF-β is able to activate STAT3, sanguinarine may regulate the HIF-1α/TGF-β/STAT3 axis to negatively control hypoxia-induced signaling (Figure 1 and Table 1).

#### 2.1.5. Tetrandrine

Accumulating evidence showed that tetrandrine possesses various anti-cancer activities, including the induction of apoptosis, the restraint of migration, invasion, and metastasis, and the reversal of drug resistance [58,59,60]. Moreover, tetrandrine can inactivate AKT and downregulate HIF-1α under normoxic conditions [42], suggesting that AKT signaling is partly responsible for HIF-1α regulation (Figure 1 and Table 1). Tetrandrine consequently reduces and increases the level of VEGF and BCL2-associated X protein (*BAX*), respectively [42], denoting the involvement of HIF-1α in the anti-angiogenic and apoptosis effects of tetrandrine in lung cancer.

### 2.2. Organosulfurs

#### 2.2.1. Diallyl Trisulfides

The metastatic potential of cancer can be enhanced by HIF-1α target genes, such as angiopoietin-like 4 (*ANGPTL4*), lysyl oxidase (*LOX*), and lysyl oxidase-like 4 (*LOXL4*) [61,62,63]. ANGPTL4 facilitates the extravasation of cancer cells [61], and LOX increases the migratory and invasive capacities of cells by activating the focal adhesion kinase (FAK)/AKT pathway [62]. In addition, LOXL4 is involved in the establishment of a metastatic niche [63]. A recent study presented that diallyl trisulfide attenuates the expression of HIF-1α, together with a decrease in ANGPTL4, LOX, and LOXL4 levels under hypoxia. Diallyl trisulfide also inhibits the expression of VEGF and EMT-associated genes such as SNAI2 [43]. They further observed that diallyl trisulfide diminishes the motility of hypoxic cancer cells in vitro and the lung metastasis of breast cancer in vivo [43]. Diallyl trisulfide inactivates AKT and extracellular signal-regulated kinase (ERK) [64,65]. ERK can stabilize HIF-1α by preventing PHD2-mediated hydroxylation of HIF-1α [66]. Therefore, diallyl trisulfide may modulate the level of HIF-1α and its target genes via both AKT-HIF-1α and ERK-HIF-1α axes (Figure 1 and Table 1).

#### 2.2.2. Sulforaphane

Sulforaphane has multitudinous anti-cancer effects. For example, sulforaphane activates caspase-3, inducing apoptotic cell death [67]. Besides, sulforaphane upregulates and downregulates miR-200c and teratocarcinoma-derived growth factor 1 (*TDGF1*), respectively, thus impeding cancer stemness [68,69]. Moreover, sulforaphane represses both expression and nuclear translocation of HIF-1α, thereby negatively regulating cell proliferation and glycolysis in bladder cancer [44]. By reducing STAT3 and HIF-1α levels, sulforaphane also diminishes VEGF expression, exerting anti-angiogenic effects in hepatocellular carcinoma [45] (Figure 1 and Table 1). STAT3 can epigenetically repress miR-200c that directly targets HIF-1α [70,71]. Thus, sulforaphane may additionally control HIF-1α expression and hypoxia-mediated signaling via the STAT3/miR-200c axis. Other miRNAs that are regulated by STAT3 may have a possibility to directly or indirectly change HIF-1α levels.

## 3. Natural Polyphenolic Compounds

### 3.1. Flavonoids

#### 3.1.1. Apigenin

**Figure 2:**
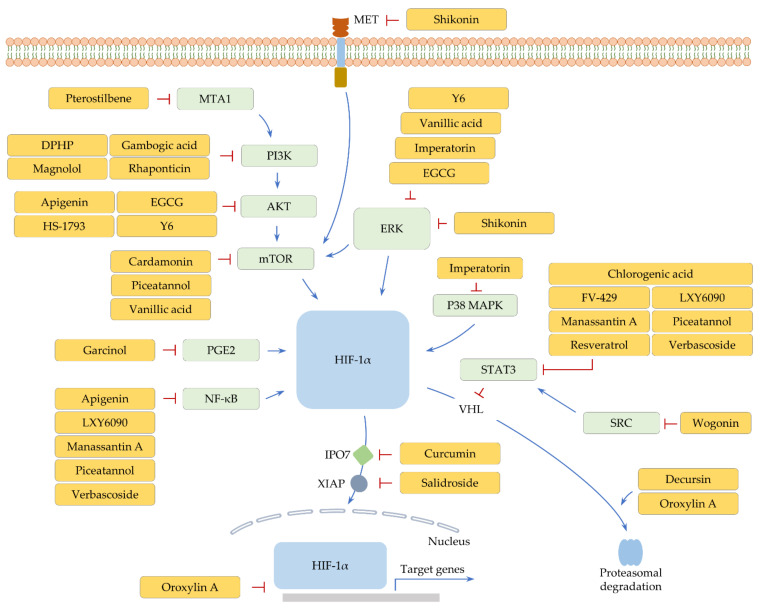
Polyphenol-mediated cellular signaling pathways that are anticipated to regulate HIF-1α. Positive regulation is pointed out by arrow lines (blue). A restrictive action is indicated by perpendicular lines (red).

The anti-cancer activity of apigenin has been evaluated in different cancer types. In osteosarcoma, apigenin diminishes the level of mitochondria membrane potential and activates caspase-3 [72]. Likewise, apigenin induces caspase-dependent apoptotic cell death and cell cycle arrest in bladder cancer cells [73]. Apigenin was observed to suppress cell migration and invasion in vitro and metastasis in vivo via downregulating the level of neural precursor cell expressed developmentally down-regulated protein 9 (*NEDD9*) in colorectal cancer [74]. It was also observed that apigenin can induce the level of inositol polyphosphate-5-phosphatase D (*INPP5D*), thus reducing the population of M2-like TAMs and eventually promoting anti-cancer immune responses in murine pancreatic cancer models [75]. In addition, the expression of HIF-1α and VEGF is downregulated by apigenin in both normoxic and hypoxic conditions. Consistently, in vivo administration of apigenin blocks angiogenesis [76]. Another study further exhibited that the levels of hypoxia-induced stemness markers such as Nanog homeobox (*NANOG*) are attenuated by apigenin in head and neck cancer [77]. It was suggested that nuclear factor-kappa B (NF-κB) transcriptionally induces HIF-1α and also stimulates HIF-1α translation [78,79]. Considering that AKT and NF-κB are inactivated by apigenin [80,81], apigenin may regulate HIF-1α levels, at least partly, through the AKT and NF-κB pathways (Figure 2 and Table 2).

#### 3.1.2. Cardamonin, Epigallocatechin-3-Gallate, and Y6

In breast cancer, cardamonin and epigallocatechin-3-gallate (EGCG) induce cell cycle arrest and apoptotic cell death [111,112]. Activation of JNK and forkhead box O3 (*FOXO3*) contributes to the upregulation of cyclin-dependent kinase inhibitor 1A (*CDKN1A*, also called p21), CDKN1B (also named as p27), and Bcl-2-like protein 11 (*BCL2L11*) following cardamonin treatment [111]. EGCG induces G2/M cell cycle arrest and inhibits cell survival by downregulating miR-25 [112]. In addition, cardamonin and EGCG can reverse the EMT process by suppressing Wnt/β-catenin signaling and DNA methyltransferase levels in breast cancer [113,114].

Moreover, both phytochemicals negatively regulate HIF-1α expression in breast cancer. Cardamonin was revealed to regulate HIF-1α expression via the mTOR pathway and repress glycolysis process by reducing the uptake of glucose and the production of lactic acid [82]. Cardamonin impedes the growth of breast cancer, along with a decrease in HIF-1α and its target genes, such as lactate dehydrogenase A (*LDHA*), in vivo [82]. EGCG diminishes HIF-1α and VEGF levels in breast cancer cells [83]. EGCG inactivates a number of cellular factors, such as AKT and ERK [115,116]. EGCG may, therefore, transcriptionally and post-transcriptionally regulate HIF-1α (Figure 2 and Table 2). It should be pointed out that EGCG can trigger autophagy-dependent survival, delaying apoptosis in consequence [117].

Further, Y6, a derivative of EGCG, downmodulates HIF-1α and ABCB1 levels in doxorubicin-resistant hepatocellular carcinoma cells. Therefore, resistant cells treated with Y6 exhibits an increase in apoptosis and sensitivity to doxorubicin [88]. Another study also demonstrated that Y6 reduces the activity of ERK and AKT and the level of HIF-1α and VEGF in hypoxic cancer cells (Figure 2 and Table 2). It was further verified that Y6 constrains the growth of hepatocellular carcinoma and angiogenesis in vivo [89].

#### 3.1.3. FV-429 and Wogonin

Therapeutic resistance and the EMT process can be suppressed by wogonin-mediated inactivation of STAT3 [118,119]. Further, it was noted that wogonin downmodulates HIF-1α levels under normoxia in gastric cancer cells (Figure 2 and Table 2). Wogonin, owing to this ability, significantly decreases several glycolysis-related genes, including LDH, succinate dehydrogenase (*SDH*), and monocarboxylate transporter 4 (*MCT4*) [87]. In their study, wogonin was shown to inhibit the proliferation of A549 lung cancer cells. However, HIF-1α expression is unaffected by wogonin in A549 cells [87]. These findings suggest that the control of HIF-1α expression by wogonin may be context-dependent. Besides, FV-429, a derivative of wogonin, diminishes the level of HIF-1α by inhibiting SRC proto-oncogene (*SRC*)/STAT3 signaling under hypoxia [84] (Figure 2 and Table 2). Therefore, FV-429 can potentiate the effectiveness of paclitaxel against ovarian cancer in vitro and in vivo [84].

#### 3.1.4. Oroxylin A

Hedgehog (Hh) signaling is activated by hypoxia and controls multiple events, such as stemness, invasion, and EMT [120]. Moreover, there is evidence that Hh signaling induces the expression of ABC transporters and DNA repair genes, triggering therapeutic resistance to several anti-cancer drugs, such as 5-fluorouracil, cisplatin, and temozolomide [121,122,123]. In glioblastoma, oroxylin A was observed to promote VHL-mediated HIF-1α degradation and limit the HIF-1α-Hh signaling pathway, thus sensitizing glioma cells to temozolomide in vitro and in vivo [85]. Interestingly, another study revealed that oroxylin A directly interacts with HIF-1α and inhibits the DNA binding property of HIF-1α, decreasing the transcription of xeroderma pigmentosum group C (*XPC*), a DNA repair gene [86] (Figure 2 and Table 2). Both oroxylin A treatment and XPC knockdown augment anti-cancer efficacy of cisplatin in vitro, indicating that oroxylin A alleviates hypoxia-induced cisplatin resistance at least partly through downregulating XPC. Indeed, oroxylin A combined with cisplatin significantly increases apoptotic cell death and inhibits the growth of lung cancer in vivo [86].

### 3.2. Lignans, Phenolic Acids, and Stilbenes

#### 3.2.1. HS-1793 and Resveratrol

STAT3 is downregulated by resveratrol administration concomitantly with an attenuation of HIF-1α as well as VEGF levels in xenograft models of lung cancer [96], indicating the inhibitory function of resveratrol in hypoxia-mediated angiogenesis. In gastric cancer, resveratrol suppresses the HIF-1α/Hh signaling axis, reversing hypoxia-driven cell invasion and the EMT process [97].

Additionally, resveratrol abates the level of HIF-1α in pancreatic stellate cells (PSCs) under hypoxia, restraining PSC activation and the secretion of various cellular factors, such as IL-6 and VEGF from PSCs [98]. The invasive capacities of pancreatic cancer cells are weakened following treatment with conditioned media (CM) from resveratrol-exposed PSCs compared to CM from hypoxia-activated PSCs [98]. These findings demonstrate that resveratrol may impair the crosstalk between cancer cells and other cellular components in the microenvironment, hence decelerating the malignant progression of cancer.

HS-1793, a resveratrol derivative, was investigated to arrest cells at the G2/M phase and induce apoptotic cell death via suppressing AKT activity [124]. Moreover, HS-1793 can exert potent anti-breast cancer effects via hindering HIF-1α-mediated transcriptional activation of VEGF expression in vitro and in vivo [90] (Figure 2 and Table 2).

#### 3.2.2. LXY6090 and Manassantin A

Manassantin A has an inhibitory role in HIF-1 transactivation activity in a dose-dependent manner [93]. In this study, evaluation of the combined effect of manassantin A and gefitinib (an epidermal growth factor receptor (*EGFR*) inhibitor) shows cooperative therapeutic effects on lung cancer in vivo [93]. This finding suggests that manassantin A sensitizes cancer cells to gefitinib, at least in part, via HIF-1α inhibition.

Besides, LXY6090, a manassantin A derivative, downregulates the expression of HIF-1α proteins via both inhibiting mRNA levels and promoting VHL-mediated degradation under hypoxia [91]. LXY6090 reduces the level of HIF-1α target genes, such as VEGF and insulin-like growth factor 2 (*IGF2*), in vitro and the growth of cancer in vivo [91]. Considering that manassantin A can inhibit NF-κB signaling and STAT3 activation [125,126], manassantin A and its derivative may transcriptionally as well as post-transcriptionally block HIF-1α expression via the NF-κB and STAT3 signaling pathways (Figure 2 and Table 2). Further investigation is desired to unveil the mechanisms of anti-cancer action of these phytochemicals.

#### 3.2.3. Magnolol

Magnolol can negatively control HIF-1α expression by interfering with the PI3K/AKT/mTOR pathway under hypoxia, hence suppressing angiogenesis and cancer growth in vivo [92]. In addition to this, functional characterization of magnolol shows that this compound can exert anti-cancer activity by inactivating MAPKs such as p38 MAPK [127]. These pieces of evidence suggest that magnolol may intricately control HIF-1α levels by affecting diverse cellular signaling pathways (Figure 2 and Table 2).

#### 3.2.4. Piceatannol and Vanillic Acid

Piceatannol shows an anti-cancer activity by exerting repressive effects on multiple cellular factors, such as mTOR and STAT3 [128]. Moreover, piceatannol can upregulate PMA-induced protein 1 (*PMAIP1*), thus strengthening cisplatin-induced apoptosis [129]. Further exploration of piceatannol revealed that NF-κB and HIF-1α levels are downregulated in colorectal cancer cells exposed to piceatannol-loaded nanoparticles (PNs) [94]. Not only do PNs inhibit colony formation and invasion activities of cancer cells in vitro, but PNs suppress the growth of colorectal cancer in vivo [94], suggesting that the NF-κB/HIF-1α axis partly mediates the anti-cancer effects of piceatannol (Figure 2 and Table 2). However, ABCB1 levels can be elevated by piceatannol exposure [130], suggesting the possibility of the incidence of multidrug resistance.

In colorectal cancer, vanillic acid also limits HIF-1α expression. Mechanistically, it was confirmed that HIF-1α is regulated by both mTOR and ERK pathways in hypoxic cancer cells following vanillic acid treatment [100] (Figure 2 and Table 2). Vanillic acid is capable of attenuating the synthesis of angiogenic factors, such as VEGF and erythropoietin (*EPO*). The anti-cancer properties of vanillic acid were determined by a decrease in cell proliferation in vitro and the growth of cancer in vivo as well [100].

#### 3.2.5. Pterostilbene

Metastasis-associated 1 (*MTA1*) is able to transcriptionally repress PTEN expression and consequently stimulate PI3K/AKT signaling [131]. Moreover, it was explored that MTA1 is induced by hypoxia and stabilizes HIF-1α proteins [132]. Recently, pterostilbene was demonstrated to abolish MTA1-mediated PTEN suppression, hence stimulating apoptosis and hindering cell invasion in vitro and cancer growth in vivo [133] (Figure 2 and Table 2). What is more, pterostilbene leads to a reduction of HIF-1α levels in conjunction with MTA1 downregulation [95]. In this study, pterostilbene was determined to enhance the efficacy of suberoylanilide hydroxamic acid (SAHA) [95]. This evidence suggests the feasibility of utilizing pterostilbene as an adjuvant to reduce the dose and toxic side effects of SAHA.

#### 3.2.6. Rhaponticin

Rhaponticin exhibits cytotoxic effects in both drug-sensitive and -resistant leukemia cells by stimulating apoptotic cell death [134]. Rhaponticin was also validated to inhibit the expression of fatty acid synthase in breast cancer and the PI3K/AKT/mTOR pathway in osteosarcoma [135,136]. Another study denoted that the nuclear expression of HIF-1α is suppressed by rhaponticin in fibrosarcoma cells exposed to hypoxic conditions and that rhaponticin reduces EMT-associated markers (e.g., SNAI2) and pro-angiogenic factors (e.g., VEGF) [99] (Figure 2 and Table 2).

### 3.3. Other Polyphenols

#### 3.3.1. Chlorogenic Acid

HIF-1α protein levels are notably downregulated by chlorogenic acid without changes in mRNA amounts in lung cancer cells exposed to cobalt (II) chloride (CoCl_2_), a hypoxia-mimetic agent [101]. This observation demonstrates post-transcriptional modulation of HIF-1α expression in response to chlorogenic acid. Such downregulation of HIF-1α is accompanied by VEGF decrement in vitro. It was then confirmed that chlorogenic acid subdues VEGF-induced angiogenesis in vivo [101]. Chlorogenic acid can interfere with the STAT3 pathway [137]. It is, therefore, feasible that HIF-1α proteins may be destabilized by VHL after chlorogenic acid exposure (Figure 2 and Table 2).

#### 3.3.2. Curcumin

Importin 7 (*IPO7*) performs a role in nuclear protein import and is overexpressed in cancers, including lung and colorectal cancer [138,139]. IPO7 knockdown reduces survival advantages of cancer cells by downregulating and upregulating AKT and BAX levels, respectively [138]. The depletion of IPO7 downregulates the nuclear import of ribosomal protein L4 and elicits ribosomal biogenesis stress. Tumor protein p53 (*TP53*) is activated by ribosomal biogenesis stress, ultimately inhibiting the colony formation of cancer cells [140]. Moreover, nuclear localization of glioma-associated oncogene homolog 1 (*GLI1*) is driven by IPO7, advancing cell proliferation and invasion in glioblastoma cells [141]. Of note, the nuclear translocation of HIF-1α can be mediated by IPO7 [142]. Nuclear accumulation of HIF-1α is impaired by curcumin, in company with an abatement of cellular factors associated with glucose metabolism under non-hypoxic conditions [102], suggesting the therapeutic potential of curcumin for chronic myelogenous leukemia. In addition, curcumin increases miR-22 that directly targets IPO7 in chronic myelogenous leukemia cells [102], indicating that curcumin modulates HIF-1α activity via the miR-22/IPO7 axis (Figure 2 and Table 2).

#### 3.3.3. Decursin

Proteasomal degradation of HIF-1α is prompted by decursin in lung and colorectal cancer cells under hypoxia [103] (Figure 2 and Table 2). Decursin downregulates the mRNA level of several genes, including C-X-C motif chemokine receptor 4 (*CXCR4*) and VEGF, indicating the transcriptional suppression of HIF-1α target genes. In vitro experiments showed that the treatment of lung cancer cells with decursin increases and decreases apoptosis and cell invasion, respectively. Further in vivo investigation noticeably demonstrated decursin-induced enhancement of anti-cancer immune response in the murine allograft model [103].

#### 3.3.4. DPHP, Garcinol, Imperatorin, Shikonin, and Verbascoside

DPHP can restrain the expression of PI3K and an activated form of AKT in colorectal cancer cells treated with CoCl_2_. HIF-1α levels are also reduced by DPHP, suggesting that PI3K/AKT signaling may influence the expression of HIF-1α in DPHP-treated conditions [104] (Figure 2 and Table 2). By altering HIF-1α expression levels, DPHP can cause a drop in VEGF and matrix metallopeptidase 2 (*MMP2*) levels [104], indicating anti-angiogenic and anti-invasive potencies of DPHP.

Prostaglandin E2 (PGE2) stabilizes HIF-1α proteins and facilitates their nuclear translocation [143]. In this study, pharmacological inhibition of the ERK pathway using PD98059 abolishes the effect of PGE2 on HIF-1α. ERK signaling has been identified to mediate PGE2 induction [144]. Therefore, HIF-1α can be stabilized and translocated to the nucleus via the ERK/PGE2 axis. In colorectal cancer, garcinol was noticed to target prostaglandin E synthase (*PTGES*) and thus decrease PGE2 productions [106]. Garcinol showed anti-migration and anti-angiogenesis effects, along with a reduction in HIF-1α and VEGF levels [106]. These results suggest that garcinol exerts anti-cancer activity via the PTGES/PGE2/HIF-1α axis in colorectal cancer (Figure 2 and Table 2).

Imperatorin was recognized to suppress cancer by halting cell cycle progression [145]. Moreover, imperatorin can augment the cytotoxicity of gamma-delta T cells against CD133-positive cancer via targeting myeloid cell leukemia 1 (*MCL1*), an endogenous apoptosis inhibitor [146]. It was further shown that imperatorin inhibits TGF-β-mediated ERK activation, thus hindering metastasis [147]. In colorectal cancer, imperatorin shows a repressive activity on HIF-1α in hypoxic conditions [107]. Imperatorin inhibits ERK and p38 MAPK, preventing the induction of HIF-1α target genes such as VEGF and EPO (Figure 2 and Table 2). Not surprisingly, imperatorin decreases the growth of colorectal cancer and the tissue expression of HIF-1α and VEGF in vivo [107].

Furthermore, shikonin causes the suppression of HIF-1α under hypoxia, resulting in efficient restraint of in vitro proliferation and in vivo growth of colorectal cancer cells [109]. Investigation of the level and activity of signaling molecules showed that shikonin inactivates mTOR independently of AKT, indicating that shikonin has an effect on other cellular factors to suppress mTOR-mediated HIF-1α regulation. Although the underlying mechanisms of such events remain elusive, ERK and MET proto-oncogene (*MET*) can be implicated in the control of HIF-1α levels, since both ERK and MET are rendered inactive by shikonin [148,149], and they can promote AKT-independent mTOR activation [150,151] (Figure 2 and Table 2).

The anti-cancer properties of verbascoside can be due to its suppressive effects on multiple cellular signaling pathways, such as NF-κB, STAT3, and TGF-β signaling [152,153,154]. Verbascoside adversely affects HIF-1α expression under normoxic conditions in colorectal cancer and downregulates EMT-related factors such as zinc finger E-box-binding homeobox 1 (*ZEB1*) [110]. NF-κB and STAT3 may be involved in verbascoside-induced HIF-1α inhibition (Figure 2 and Table 2). In addition, TGF-β can increase the nuclear expression of HIF-2α, activating VEGF transcription under normoxic conditions [155]. Therefore, verbascoside may also abrogate HIF-2α signaling in non-hypoxic cells.

#### 3.3.5. Gambogic Acid

The therapeutic potential of gambogic acid against cancer has been determined by its abrogative effects on NF-κB and STAT3 signaling, leading to apoptosis and anti-angiogenesis [156,157]. In addition, PI3K/AKT/mTOR signaling is deactivated in gambogic acid-treated cancer cells [158]. In multiple myeloma, gambogic acid restrains hypoxia signaling [105]. Under hypoxia, the induction of HIF-1α and VEGF is significantly attenuated by gambogic acid, and PI3K/AKT/mTOR signaling is engaged in HIF-1α regulation (Figure 2 and Table 2). Further, it was presented that the growth of multiple myeloma is impaired in the gambogic acid-treated xenografts [105]. However, it is noteworthy that caspase activation can be detained by gambogic acid-induced autophagy [156].

#### 3.3.6. Salidroside

Oxaliplatin resistance can be advanced by a number of cellular factors and events, such as XIAP and EMT. Knockdown of XIAP restores the sensitivity of resistant cancer cells to oxaliplatin. ZEB1 is upregulated in oxaliplatin-resistant cells, and its silencing increases oxaliplatin-induced apoptotic cell death [159,160]. Besides, pharmacological inhibition of HIF-1α enhances the anti-cancer potency of oxaliplatin, demonstrating the association of hypoxia with oxaliplatin resistance [161]. Recent research showed that salidroside decreases HIF-1α and EMT factors such as ZEB1 under hypoxia. Thus, salidroside can enhance the oxaliplatin sensitivity of hepatocellular carcinoma cells in vitro and in vivo [108]. Salidroside was reported to downregulate XIAP, and XIAP was interestingly discerned to promote the nuclear retention of HIF-1α and subsequently escalate the level of HIF-1α responsive genes [162,163]. Therefore, the XIAP/HIF-1α axis may be one of the signaling axes accountable for the resistance-alleviating effect of salidroside (Figure 2 and Table 2).

## 4. Terpene Phytochemicals

### 4.1. Monoterpenes

#### 4.1.1. Perillyl Alcohol

**Figure 3:**
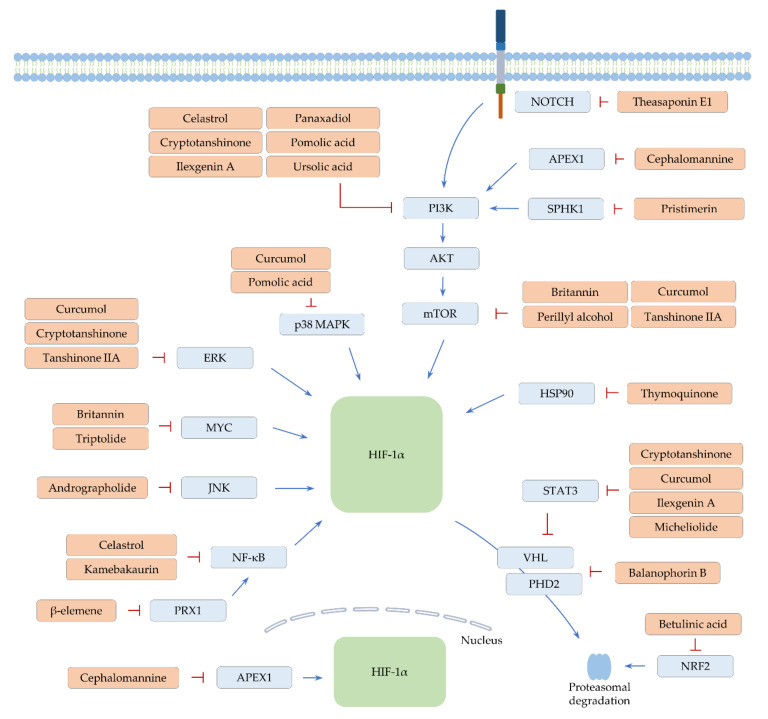
Proposed mechanisms by which terpenes regulate HIF-1α. Positive modulation is denoted by arrow lines (blue). Perpendicular lines designate an inhibitory effect (red).

In vitro reporter assays showed that perillyl alcohol efficiently blocks the transcriptional activity of HIF-1α in several types of cancer cells treated with CoCl_2_ [164]. Moreover, perillyl alcohol efficiently reduces mTOR activation and the level of HIF-1α and VEGF proteins under CoCl_2_-mediated hypoxic conditions [164], suggesting that perillyl alcohol negatively regulates angiogenic signaling via the mTOR/HIF-1α pathway (Figure 3 and Table 3). In vivo administration of perillyl alcohol remarkably arrests the growth of colorectal cancer with a decrease in VEGF levels in the serum [164].

#### 4.1.2. Thymoquinone

The screening of 502 natural compounds identified thymoquinone as one of the HIF-1α inhibitors [183]. Treatment with thymoquinone represses HIF-1α expression under hypoxia in renal cancer cells. Thus, thymoquinone downregulates various HIF-1α target genes involved in glycolysis, angiogenesis, and metastasis [183]. Heat shock protein 90 (HSP90) has been known to physically interact with and protect HIF-1α from proteasome-mediated degradation independently of VHL [188]. Thymoquinone was observed to destabilize HIF-1α by hindering the interaction of HIF-1α with HSP90, ultimately promoting the apoptosis of hypoxic cancer cells [183] (Figure 3 and Table 3).

### 4.2. Sesquiterpenes

#### 4.2.1. β-Elemene and Micheliolide

A decrease in HIF-1α and GLUT1 expression was detected in lung cancer xenografts treated with β-elemene [165]. The administration of β-elemene sensitizes lung cancer to radiotherapy, demonstrating that β-elemene can improve radiotherapy efficiency by impeding hypoxia-mediated glycolysis. Another study demonstrated that the treatment of hypoxic cancer cells with β-elemene downregulates the expression of peroxiredoxin 1 (*PRX1*), a mediator of NF-κB activation [166]. Restraint of the PRX1/NF-κB pathway contributes to an attenuation of HIF-1α levels in lung cancer (Figure 3 and Table 3). Such HIF-1α inhibition further suppresses hypoxia-mediated induction of monocyte chemoattractant protein-1 (*MCP1*), ultimately restricting the infiltration of TAMs into the tumor microenvironment and enhancing the effect of radiotherapy [166].

Micheliolide also sensitizes lung cancer cells to irradiation, owing to its prohibitory action on HIF-1α. Notably, micheliolide was confirmed to facilitate HIF-1α degradation [177]. Although it is required to uncover molecular mechanisms accounting for micheliolide-mediated HIF-1α degradation, STAT3 can be involved in HIF-1α regulation, since its activity is inhibited by micheliolide [189] (Figure 3 and Table 3).

#### 4.2.2. Britannin

Tumor necrosis factor (*TNF*) can induce and stabilize PD-L1, driving cancer immune evasion [190,191]. PD-L1 is also known as one of the HIF-1α targets [17], and TNF has been shown to induce HIF-1α [192]. These findings suggest that the TNF-HIF-1α pathway plays a part in controlling PD-L1 expression. More recently, britannin was recognized to exhibit anti-colorectal cancer effects via downmodulating TNF-induced PD-L1 expression [170]. Britannin interrupts mTOR signaling and inhibits MYC proto-oncogene (*MYC*), as well as HIF-1α expression in TNF-treated colorectal cancer cells [170]. Since MYC is able to post-transcriptionally induce HIF-1α proteins [193], britannin may abrogate the effect of TNF on HIF-1α via affecting mTOR and MYC, thus leading to a decline in PD-L1 (Figure 3 and Table 3). The growth of cancer is inhibited by britannin, together with a reduction of PD-L1 and VEGF in vivo [170], proposing the therapeutic potential of britannin towards colorectal cancer.

#### 4.2.3. Curcumol

Similarly, curcumol downregulates HIF-1α and PD-L1 expression under hypoxia in hepatocellular carcinoma cells [174]. Curcumol adversely affects the activity of STAT3, mTOR, and MAPKs (e.g., ERK and p38 MAPK), implying the feasibility of involvement of these signaling factors in HIF-1α regulation [174] (Figure 3 and Table 3). Curcumol enhances T cell-mediated lysis of hepatocellular carcinoma cells in vitro and retards the growth of cancer in vivo [174]. Combination therapy with PD-L1 inhibitors and other therapeutic approaches (e.g., chemotherapy and immunotherapy) has been suggested to reinforce anti-cancer responses [194]. Therefore, the development of effective curcumol-based combination strategies may enhance therapeutic responses.

### 4.3. Diterpenes

#### 4.3.1. Andrographolide

The anti-cancer action of andrographolide is through multiple mechanisms, including the suppression of ERK activity and cyclooxygenase 2 (COX2) expression [195,196]. In hepatocellular carcinoma, andrographolide diminishes the level of HIF-1α and VEGF in vitro and in vivo [167]. In this study, it was additionally noticed that inhibition of JNK using a pharmacological inhibitor, SP600125, nullifies the effect of andrographolide on HIF-1α expression [167] (Figure 3 and Table 3). The role of JNK in regulating HIF-1α expression is inconclusive, because JNK has been reported to repress or advance HIF-1α levels [197,198], proposing the requirement of more investigation on the relationship between JNK and HIF-1α.

#### 4.3.2. Cephalomannine

Apurinic/apyrimidinic endodeoxyribonuclease 1 (*APEX1*) advances malignant properties, such as proliferation, invasion, and angiogenesis through activating, for example, PI3K/AKT and Notch signaling [199,200]. In addition, APEX1 interacts with HIF-1α and positively affects the transcription activity of HIF-1α under hypoxia [201]. Recently, it was demonstrated that cephalomannine inhibits the interaction of APEX1 and HIF-1α, resulting in the attenuation of cell viability and migration of lung cancer cells under hypoxic conditions [172] (Figure 3 and Table 3). In their study, cephalomannine was also confirmed to impede the growth of lung cancer with a reduction of several HIF target genes such as MMP2 in xenograft models [172].

#### 4.3.3. Cryptotanshinone and Kamebakaurin

The anti-colorectal cancer effects of cryptotanshinone can be caused by the downregulation of HIF-1α and VEGF levels in vivo [173]. Cryptotanshinone exerts a negative influence on the PI3K/AKT/mTOR signaling in vitro [173], implying the participation of this signaling pathway in HIF-1α regulation. Apart from this, cryptotanshinone can exert its anti-cancer activity via inactivating STAT3 and ERK [202,203]. Thus, it can be speculated that multiple cellular factors affected by cryptotanshinone coordinately modulate HIF-1α signaling (Figure 3 and Table 3).

Kamebakaurin is capable of inhibiting the activity of NF-κB and blocks the induction of anti-apoptotic genes controlled by NF-κB. Thus, cancer cells treated with kamebakaurin become susceptible to apoptosis [204]. Further, kamebakaurin causes downregulation of HIF-1α and its target gene levels, impairing cancer progression in vitro and in vivo [176]. Reduction of HIF-1α proteins took place without an alteration of mRNA levels and protein stability in CoCl_2_-treated colorectal cancer cells [176], suggesting the ability of kamebakaurin to inhibit translation of HIF-1α mRNA. The molecular mechanisms behind HIF-1 regulation are unknown. However, it is conceivable that NF-κB can be one of the critical mediators in kamebakaurin-mediated downregulation of HIF-1 (Figure 3 and Table 3).

#### 4.3.4. Tanshinone IIA

Accumulating evidence has demonstrated the efficacy of tanshinone IIA against cancer. For instance, lipid peroxidation and ferroptotic cell death can be induced by tanshinone IIA in gastric cancer [205]. In addition, tanshinone IIA can trigger caspase activation and cell cycle arrest in breast cancer cells, along with a forceful inhibition of ERK, mTOR, and protein kinase C activities [206]. Evidence from another study showed that tanshinone IIA negatively modulates both HIF-1α and VEGF levels via hampering the mTOR signaling pathway under normoxic and hypoxic conditions in vitro [181] (Figure 3 and Table 3). Tanshinone IIA indeed showed anti-angiogenesis and cancer growth suppression potency in vivo [181].

#### 4.3.5. Triptolide

Diverse cellular events, including apoptosis, cellular senescence, and EMT, are influenced by triptolide [207,208,209], connoting its therapeutic benefit against cancer. Further, triptolide can attain its anti-pancreatic cancer activity via imposing limits on HIF-1α expression in vitro and in vivo [184]. MYC is downregulated by triptolide at the transcription level [184], suggesting the high likelihood of MYC involvement in HIF-1α regulation (Figure 3 and Table 3).

### 4.4. Triterpenes

#### 4.4.1. Balanophorin B

In hepatocellular carcinoma, the expression of HIF-1α and its target genes LDHA and hexokinase 2 (*HK2*) is inhibited by balanophorin B under hypoxia, resulting in the suppression of glycolysis in vitro. Moreover, balanophorin B retards cancer growth without inducing normal tissue toxicity in vivo [168]. In their study, balanophorin B was noticed to augment the expression of VHL and PHD2 without a change in HIF-1α mRNA levels, suggesting post-transcriptional regulation of HIF-1α [168] (Figure 3 and Table 3). Balanophorin B deserves further investigation to explore signaling pathways associated with HIF-1α regulation and to uncover other mechanisms by which balanophorin B exerts anti-cancer activity.

#### 4.4.2. Betulinic Acid

The anti-cancer activity of betulinic acid occurs through the regulation of several events, such as apoptosis and metastasis [210,211]. Of note, betulinic acid can control stemness and drug resistance [212,213], suggesting that betulinic acid is one of the potential candidate agents for cancer treatments. Moreover, it was presented that HIF-1α accumulation is weakened by betulinic acid, leading to a diminution of HIF-1α responsive genes such as VEGF and GLUT1 in hypoxic cervical cancer cells [169]. In this study, it was suggested that betulinic acid inhibits HIF-1α accumulation by activating proteasome. Such proteasome activation may be mediated by NRF2, which can upregulate the level of several proteasome genes and is activated by betulinic acid [214,215] (Figure 3 and Table 3). Further investigation is required to disclose the mechanisms of proteasome activation by betulinic acid.

#### 4.4.3. Celastrol

Celastrol has been determined to adversely affect cancer progression and metastasis in multiple types of cancer [216]. The findings from mechanism studies revealed that celastrol is able to inhibit VEGF expression, PI3K/AKT/mTOR, and NF-κB signaling [217]. In glioblastoma, celastrol inhibits cell viability as well as the migratory and invasive capacities of cancer cells in vitro [171]. The findings from in vivo experiments showed that celastrol limits glioblastoma infiltration. Celastrol suppresses angiogenesis, vasculogenic mimicry formation (VMF), and the level of angiogenesis- and VMF-promoting factors, such as VEGF, EPH receptor A2 (*EPHA2*), and cadherin 5 (*CDH5*, also called VE-cadherin). Mechanistically, celastrol blocks the PI3K/AKT/mTOR pathway and reduces the expression of HIF-1α [171]. Both EPHA2 and CDH5 can be positively regulated by HIF-1α [218,219]. Therefore, celastrol may downregulate angiogenesis and VMF via the PI3K/AKT/mTOR/HIF-1α pathway in glioblastoma (Figure 3 and Table 3).

#### 4.4.4. Ilexgenin A and Panaxadiol

Ilexgenin A has been shown to repress melanoma via inducing cell cycle arrest and exerting anti-hepatocellular carcinoma activity by inhibiting the PI3K and STAT3 pathways [220,221]. Of note, the combination of ilexgenin A and sorafenib has synergistic effects on hepatocellular carcinoma growth [221]. This finding can provide a possibility to develop a novel treatment strategy against hepatocellular carcinoma. Moreover, ilexgenin A inhibits HIF-1α expression, consequently downregulating sterol regulatory element-binding transcription factor 1 (*SREBF1*) expression and lipid accumulation in colorectal cancer cells [175] (Figure 3 and Table 3). Treatment of cancer cells with ilexgenin A reduces cell viability and mRNA levels of β-catenin and TNF, suggesting that ilexgenin A acts by disturbing multiple signaling events. Ilexgenin A attenuates colitis-induced carcinogenesis in conjunction with a reduction of HIF-1α and SREBF1 levels in vivo [175]. These findings demonstrate that ilexgenin A possesses an anti-carcinogenic activity and impedes the progression of cancer.

In colorectal cancer cells, panaxadiol suppresses HIF-1α expression via the PI3K/AKT pathway under hypoxia [178] (Figure 3 and Table 3). In addition, panaxadiol was noticed to inhibit PD-L1, thereby restoring the cytotoxic activity of T cells against cancer cells in vitro. As expected, the growth of colorectal cancer is effectively hampered by panaxadiol in vivo [178]. Findings from this study may provide the basis for developing panaxadiol as a HIF-1α/PD-L1 inhibitor to treat colorectal cancer.

#### 4.4.5. Pomolic Acid

Increasing evidence demonstrated that pomolic acid exerts anti-cancer activity by inducing apoptotic cell death and inhibiting invasion and metastasis [222,223,224]. Pomolic acid also mitigates multidrug resistance via inhibiting ABCB1 activity [225]. In breast cancer, pomolic acid was discerned to abolish the stimulatory effect of EGF on HIF-1α and VEGF expression [179]. It was additionally indicated that the negative effects of pomolic acid on EGF-induced HIF-1α and VEGF accumulation is due to its repressive action on PI3K/AKT/mTOR signaling and p38 MAPK activity [179] (Figure 3 and Table 3).

#### 4.4.6. Pristimerin

In cancer cells, sphingosine kinase 1 (*SPHK1*) is activated by reactive oxygen species (ROS) and increases AKT activity under hypoxia, eventually stabilizing HIF-1α proteins [226]. Lately, hypoxia-induced SPHK1 was noticed to be significantly suppressed by pristimerin in prostate cancer cells, resulting in a downregulation of AKT activity and HIF-1α expression [180] (Figure 3 and Table 3). Unsurprisingly, cell viability and VEGF levels are restrained by pristimerin under hypoxia [180]. SPHK1 is also known to upmodulate HIF-2α expression under hypoxia [227]. Thus, it seems probable that pristimerin controls hypoxia signaling via limiting both HIF-1α and HIF-2α.

#### 4.4.7. Theasaponin E1

The Notch pathway generally prevents apoptotic cell death through activating AKT [228]. Inhibition of this pathway leads to the enhancement of anti-cancer drug efficacy by promoting growth inhibition, cell cycle arrest, and apoptotic cell death [229]. Theasaponin E1 showed effective growth inhibition in several types of cancer, including breast, uterus, and gastric cancer [230]. Moreover, theasaponin E1 depresses Notch signaling, AKT activation, and HIF-1α levels, suggesting that HIF-1α can be partly regulated by the Notch/AKT axis in theasaponin E1-treated cells [182] (Figure 3 and Table 3). Theasaponin E1 can inhibit angiogenesis and cause apoptosis via activating caspases, cell cycle arrest, and migration in cisplatin-resistant ovarian cancer cells. In contrast, low cytotoxicity of theasaponin E1 was observed in normal cells [182], indicating its selective cytotoxicity towards ovarian cancer cells.

#### 4.4.8. Ursolic Acid

Multiple studies have shown that ursolic acid effectively alleviates therapeutic resistance in cancer. Oxaliplatin-induced apoptosis is enhanced by ursolic acid in colorectal cancer cells [231]. Ursolic acid reverses paclitaxel resistance by upregulating miR-149–5p, a tumor-suppressive miRNA, in breast cancer cells [232]. In addition, ursolic acid can counter therapeutic resistance, owing to its capability to inhibit HIF-1α levels. The expression of both HIF-1α and ABCB1 is reduced by ursolic acid in hypoxic colorectal cancer cells. Ursolic acid thereby sensitizes hypoxic cells to 5-fluorouracil and augments 5-fluorouracil-induced apoptosis [185]. Another study demonstrated that radio-resistance of HIF-1α-overexpressing lung cancer cells is weakened by ursolic acid treatment [186]. Moreover, ursolic acid can deactivate PI3K/Akt signaling, bringing about downregulation of HIF-1α and ABCG2 levels in hypoxic ovarian cancer stem cells [187] (Figure 3 and Table 3). Further, ursolic acid downmodulates the expression of stemness factors, such as NANOG, CD44, octamer-binding protein 4 (*OCT4*), and prominin-like protein 1 (*PROM1*, also named CD133), and enhances the efficacy of cisplatin under hypoxia [187]. These results imply that ursolic acid may strengthen the anti-cancer effect of other therapeutic agents by altering drug efflux and cancer stemness.

## 5. Conclusions

Accumulated evidence introduced here showed that phytochemicals can be used as potent HIF-1α inhibitors and that the suppression of HIF-1α can reinforce the efficiency of cancer treatments, such as chemotherapy and radiation therapy. These findings suggest that phytochemicals are substantial sources of new HIF-1α inhibitors. Since Y6 and ursolic acid can restrict HIF-1α expression in both normoxic and hypoxic conditions, they may potentially block HIF-1α-related signaling in cancer. Furthermore, verbascoside and pristimerin can act as dual HIF-1α and HIF-2α inhibitors. In this regard, the development of phytochemical-based treatment has a considerable potentiality to improve therapeutic benefits.

Even though phytochemicals possess anti-cancer properties, cellular protection mechanisms, such as autophagy and the induction of efflux pumps, can be actuated by them. These findings imply the inevitable emergence of resistance towards phytochemicals. Accordingly, the development of an optimal combination of phytochemicals with other autophagy inhibitors can be one of the beneficial strategies against cancer. A deep understanding of molecular mechanisms is necessary to move phytochemicals from preclinical tests to clinical trials and then clinical practice in the future.

## Figures and Tables

**Table 2:** The list of polyphenolic compounds that inhibit HIF-1α in cancer (alphabetical order).

Compound	Compound Class	Type of Cancer	In Vitro Testing (Effective Concentrations, Cell Line, Culture Condition (Normoxia/Hypoxia))	In Vivo Experiment Model (Dose and Administration Route)	Ref.
Polyphenols (Flavonoids)					
Apigenin	Trihydroxyflavone	Prostate, Ovarian, Colon, and Breast cancer	10–40 μM (human PC-3, DU145, MCF-7, HCT-8, LNCaP cell lines), hypoxia (1% O_2_)	PC-3 and OVCAR-3-bearing CAM assay (7.5–20 μM), Matrigel plug assays (15–20 μM)	[76]
		Head and Neck cancer	20–40 μM (human HN-30 cell line), hypoxia (0.5–1% O_2_)	-	[77]
Cardamonin	Chalcone	Breast cancer	20–40 μM (human MDA-MB-231 cell line), hypoxia (CoCl_2_)	Subcutaneous injection of MDA-MB-231 cells (3 mg/kg, intra-peritoneal)	[82]
Epigallocatechin-3-gallate (EGCG)	Flavanol	Breast cancer	5–20 μM (human MCF-7 cell line), normoxia	-	[83]
FV-429	Wogonin derivative	Ovarian cancer	5–20 μM (human SK-OV-3 and A2780 cell lines), hypoxia (1% O_2_)	Subcutaneous injection of A2780 cells (10 mg/kg)	[84]
Oroxylin A	Dihydroxyflavone	Glioblastoma	25–20 μM (human U251 cell line), 10–20 μM (rat C6 cell line), hypoxia (1% O_2_)	Intracranial transplantations of U251 cells or subcutaneous injections of mouse GL261 cell line (300 mg/kg, oral)	[85]
		Lung cancer	50 μM (human H460, A549, 95D, PC9, HCC827 and H1975 cell lines), hypoxia (1% O_2_)	Subcutaneous injections of H460 cells (50 mg/kg, oral)	[86]
Wogonin	Hydroxyflavone	Gastric cancer	20–100 μM (human SGC-7901 cell line), normoxia	-	[87]
Y6	EGCG derivative	Hepatocellular carcinoma	10–15 μM (doxorubicin-resistant human BEL-7404 cell line), normoxia	-	[88]
		Hepatocellular carcinoma	10 μg/mL (human SMMC-7721 cell line), hypoxia (1% O_2_)	CAM model for angiogenesis assay (200–500 μg/mL), Subcutaneous injections of HepG2 cells (55 mg/kg, oral)	[89]
Polyphenols (Lignans, Phenolic Acids, and Stilbenes)					
HS-1793	Resveratrol analogue	Breast cancer	12.5–50 μM (human MCF-7 and MDA-MB-231 cell lines), hypoxia (1% O_2_)	Subcutaneous injections of MDA-MB-231 cells (5–10 mg/kg, intraperitoneal)	[90]
LXY6090	Manassantin A derivative	Breast cancer	0.4–10 nM (human T47D, MCF-7, and MX-1 cell lines), hypoxia (1% O_2_)	Subcutaneous injections of MX-1 cells (25–100 mg/kg, oral)	[91]
Magnolol	Lignan	Bladder cancer	1–10 μM (human T24 cell line), hypoxia (1% O_2_)	CAM model of T24 cells (1–10 μM), Matrigel plug assays (25–75 μg), Subcutaneous injections of T24 cells (2–10 mg/kg, intraperitoneal)	[92]
Manassantin A	Lignan	Lung cancer	0.01–10 μM (luciferase-reporter assay using human embryonic kidney 293T cells), hypoxia (1% O_2_)	Lewis lung carcinoma allografts (5 mg/kg, intraperitoneal)	[93]
Piceatannol	Stilbene	Colorectal cancer	7.5 μg/mL of piceatannol-loaded nanoparticles (PNs) (human CaCo-2 and HT-29 cell lines), normoxia	Colitis-associated colorectal cancer mouse model (40 mg/kg)	[94]
Pterostilbene	Stilbene	Prostate cancer	50 μM (human LNCaP and PC3M cell lines), normoxia	Pten-null mouse model (10 mg/kg, intraperitoneal)	[95]
Resveratrol	Stilbene	Lung cancer	-	Orthotopic injections of A549 cells (250 mg/kg, intragastric)	[96]
		Gastric cancer	12.5–100 μM (human SGC-7901 cell line), hypoxia (3% O_2_)	-	[97]
		Pancreatic cancer	50 μM (human pancreatic stellate cells from normal tissues), hypoxia (3% O_2_)	KPC mouse model of pancreatic cancer (50 mg/kg)	[98]
Rhaponticin	Stilbene	Fibrosarcoma	25–100 μM (human HT1080 cell line), hypoxia (CoCl_2_)	-	[99]
Vanillic acid	Phenolic acid	Colorectal cancer	3–30 μM (human HCT-116 cell line), hypoxia (1% O_2_, CoCl_2_)	Subcutaneous injections of HCT-116 cells (10–30 mg/kg, oral)	[100]
Other Polyphenols					
Chlorogenic acid	Tannin	Lung cancer	2–10 μM (human A549 cell line), hypoxia (1% O_2_, CoCl_2_)	Matrigel plug assays (10 μM)	[101]
Curcumin	Diarylheptanoid	Chronic myelogenous leukemia	20 μM (human K526 cell line), normoxia	-	[102]
Decursin	Pyranocoumarin	Lung and Colorectal cancer	10–50 μM (human A549 and HCT-116 cell lines), hypoxia (1% O_2_, CoCl_2_)	Lewis lung carcinoma allografts (10 mg/kg, intraperitoneal)	[103]
DPHP	Alpinoid c (diarylheptanoid) derivative	Colorectal cancer	3.5–14 μM (human COLO205 cell line), hypoxia (CoCl_2_)	CAM model for angiogenesis assay (3.5–14 μM)	[104]
Gambogic acid	Xanthone	Multiple myeloma	0.1–0.2 μM (human U266 cell line), hypoxia (1% O_2_)	Subcutaneous injections of U266 cells (2–4 mg/kg, intravenous)	[105]
Garcinol	Polyisoprenylated benzophenone	Colorectal cancer	20–60 μM (human HT-29 cell line), normoxia	-	[106]
Imperatorin	Furanocoumarin	Colorectal cancer	50–150 μM (human HCT-116 cell line), hypoxia (1% O_2_, CoCl_2_)	Subcutaneous injections of HCT-116 cells (50–100 mg/kg, oral)	[107]
Salidroside	Phenylethanoid	Hepatocellular carcinoma	100 μM (human PLC/PRF/5, SMMC-7721, and HepG2 cell lines), hypoxia (1% O_2_)	Subcutaneous or orthotopic injections of PLC/PRF/5 cells (60 mg/kg, intragastric)	[108]
Shikonin	Naphthoquinone	Colorectal cancer	1–10 μM (human SW-620 and HCT-116 cell lines), hypoxia (1% O_2_)	Subcutaneous injections of HCT-116 cells (2–10 mg/kg, oral)	[109]
Verbascoside	Phenylethanoid glycoside	Colorectal cancer	50–150 μM (human HT-29 cell line), normoxia	-	[110]

**Table 3:** The list of terpene compounds restricting HIF-1α in cancer (alphabetical order).

Compound	Compound Class	Type of Cancer	In Vitro Testing (Effective Concentrations, Cell Line, Culture Condition (Normoxia/Hypoxia)	In Vivo Experiment Model (Dose and Administration Route)	Ref.
β-elemene	Sesquiterpene	Lung cancer	-	Subcutaneous injections of A549 cells (45 mg/kg)	[165]
		Lung cancer	-	Lewis lung carcinoma allografts (45 mg/kg, intraperitoneal)	[166]
Andrographolide	Diterpene	Hepatocellular carcinoma	25–50 μM (human Hep3B and HepG2 cell lines), normoxia	Subcutaneous injections of Hep3B cells (10 mg/kg, intraperitoneal)	[167]
Balanophorin B	Triterpene	Hepatocellular carcinoma	25–50 μM (human Huh-7 and HepG2 cell lines), hypoxia (1% O_2_)	Subcutaneous injections of HepG2 cells (50–100 mg/kg, oral)	[168]
Betulinic acid	Triterpene	Cervical cancer	3–30 μM (human HeLa cell line), hypoxia (1% O_2_)	-	[169]
Britannin	Sesquiterpene	Colorectal cancer	1–10 μM (human HCT-116 cell line), normoxia	Subcutaneous injections of HCT-116 cells (5–15 mg/kg, oral)	[170]
Celastrol	Triterpene	Glioblastoma	0.25–1 μM (human U87 and U251 cell lines), normoxia	Orthotopic injections of U87 cells (0.5–2 mg/kg, intraperitoneal)	[171]
Cephalomannine	Diterpene	Lung cancer	0.025–0.1 μM (human A549 and H460 cell lines), hypoxia (1% O_2_)	Subcutaneous injections of H460 cells (0.4 mg/kg, intraperitoneal)	[172]
Cryptotanshinone	Diterpene	Colorectal cancer	5–20 μM (mouse CT26 cell line), normoxia	Subcutaneous injections of CT26 cells (20–80 mg/kg, oral)	[173]
Curcumol	Sesquiterpene	Hepatocellular carcinoma	3–30 μM (human Hep3B cell line), hypoxia (1% O_2_)	Subcutaneous injections of Hep3B cells (3–30 mg/kg, oral)	[174]
Ilexgenin A	Triterpene	Colorectal cancer	25–50 μM (human HT-29 and HCT-116 cell lines), hypoxia (1% O_2_)	Colitis-associated colorectal cancer mouse model (20 mg/kg)	[175]
Kamebakaurin	Diterpene	Colorectal cancer	10–30 μM (human HCT-116 cell line), hypoxia (CoCl_2_)	Subcutaneous injections of HCT-116 cells (15–50 mg/kg, oral)	[176]
Micheliolide	Sesquiterpene	Lung cancer	5–20 μM (human H1299 and Calu-1 cell lines), hypoxia (1% O_2_)	-	[177]
Panaxadiol	Triterpene	Colorectal cancer	10 μM (human HCT-116 cell line), hypoxia (1% O_2_)	Subcutaneous injections of HCT-116 cells (10–30 mg/kg, oral)	[178]
Perillyl alcohol	Monoterpene	Cervical and Colorectal cancer, Hepatocellular carcinoma	50–200 μM (human HCT-116, HeLa, and SK-Hep1 cell lines), hypoxia (1% O_2_, CoCl_2_)	Subcutaneous injections of HCT-116 cells (50–100 mg/kg, oral)	[164]
Pomolic acid	Triterpene	Breast cancer	1–10 μM (human MCF-7 and MDA-MB-231 cell lines), hypoxia (CoCl_2_)	-	[179]
Pristimerin	Triterpene	Prostate cancer	1 μM (human PC-3, DU145, and LNCaP cell lines), hypoxia (1% O_2_)	-	[180]
Tanshinone IIA	Diterpene	Breast cancer	2.5–20 μM (human MCF-7 and MDA-MB-231 cell lines), hypoxia (1% O_2_)	Subcutaneous injections of MDA-MB-231 cells (50 mg/kg, intraperitoneal)	[181]
Theasaponin E1	Triterpene	Ovarian cancer	1–5 μM (human OVCAR-3 and A2780/CP70 cell lines), normoxia	CAM model of OVCAR-3 cells (4 μM)	[182]
Thymoquinone	Monoterpene	Renal cancer	5–10 μM (human Caki-1, Caki-2, and A498 cell lines), hypoxia (1% O_2_)	-	[183]
Triptolide	Diterpene	Pancreatic cancer	55–140 μM (human SW1990 cell line), normoxia	Subcutaneous injections of SW1990 cells (0.2–0.8 mg/kg, intraperitoneal)	[184]
Ursolic acid	Triterpene	Colorectal cancer	20–40 μM (human RKO, LoVo, and SW480 cell lines), hypoxia (1% O_2_)	-	[185]
		Lung cancer	50–80 μM (human H1299 cell line), normoxia	-	[186]
		Ovarian cancer	6.5–65 μM (spheroid cultures of human SKOV3 cell line), hypoxia (1% O_2_)	-	[187]

## Supplementary Materials

Supplementary Materials can be found at https://www.mdpi.com/article/10.3390/ijms22189819/s1. Table S1. The structure, source, and clinical trial status of phytochemicals.
