# Supplementary material for: Anti-Cancer Activity of Phytochemicals Targeting Hypoxia-Inducible Factor-1 Alpha
Source: Int J Mol Sci. 2021 Sep 10;22(18):9819. doi: 10.3390/ijms22189819 (PMC8467787; doi:10.3390/ijms22189819)
Supplement: Supplementary file 1 [file ijms-22-09819-s001.zip › ijms-1343737-supplementary.pdf]

**Anticancer Activity of Phytochemicals Targeting Hypoxia-Inducible Factor-1 Alpha**

by Yun et al.

**Table S1.** The structure, source, and clinical trial status of phytochemicals.

| Phytochemical (NCT number, Condition/Disease, Recruitment Status if applicable) | Structure | Source | Ref. |
|---|---|---|---|
| | **Alkaloids** | | |
| Berberine * (NCT02226185, colorectal cancer, completed) | 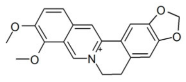 | Berberis species | [1] |
| Cyclopamine tartrate | 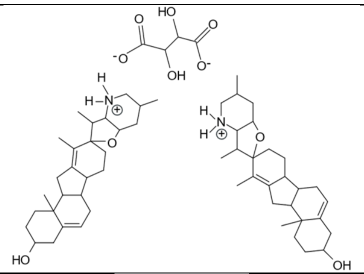 | Derivative of cyclopamine, which is from *Fritillaria* and *Veratrum* plant | [2,3] |
| Dictamnine | 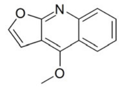 | The roots of *Dictamnus dasycarpus Turcz* | [4] |
| Nuciferine | 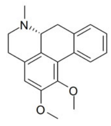 | *Nymphaea caerulea* and *Nelumbo nucifera* | [5] |
| Sanguinarine | 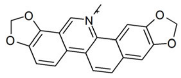 | *Sanguinaria canadensis*, *Chelidonium majus*, and *Argemone Mexicana* | [6] |
| Tetrandrine | 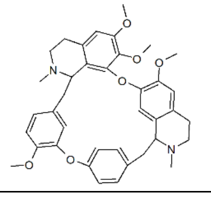 | The roots of *Stephania tetrandra* | [7] |
| | **Organosulfurs** | | |
| Diallyl trisulfide | 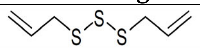 | Garlic (*Allium sativum L*) | [8] |
| Sulforaphane * (NCT03232138, lung cancer, recruiting) (NCT03665922, prostate cancer, recruiting) (NCT00982319, breast cancer, completed) | 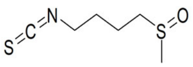 | Vegetables, such as cauliflower and brussel sprouts | [9] |
| | **Polyphenols (Flavonoids)** | | |

| | | | |
|---|---|---|---|
| Apigenin | 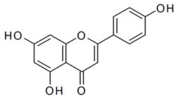 | Dried flowers of *Matricaria chamomilla* | [10] |
| Cardamonin | 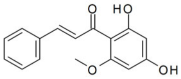 | *Alpinia katsumadai* | [11] |
| Epigallocatechin-3-gallate (EGCG) * (NCT02891538, colorectal cancer, recruiting) (NCT00676780, prostate cancer, completed) | 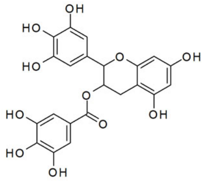 | Green tea | [12] |
| FV-429 | 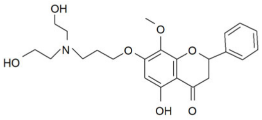 | A derivative of wogonin, which is from *Scutellaria baicalensis Georgi* | [13] |
| Oroxylin A | 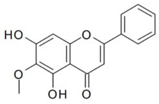 | *Scutellaria baicalensis Georgi* | [14] |
| Wogonin | 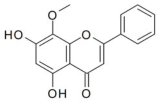 | *Scutellaria baicalensis Georgi* | [15] |
| Y6 | 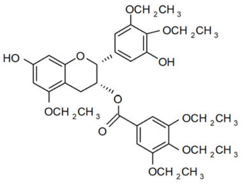 | A derivative of Epigallocatechin-3-gallate (EGCG) | [16] |
| **Polyphenols (Lignans, Phenolic Acids, and Stilbenes)** | | | |
| HS-1793 | 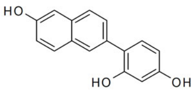 | An analogue of resveratrol, which is from grapes and peanuts | [17] |
| LXY6090 | 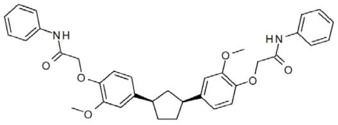 | A derivative of Manassantin A, which is from *Saururuscernuus* L. | [18] |
| Magnolol | 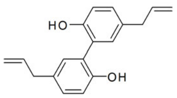 | *Magnolia officinalis* | [19] |
| Manassantin A | 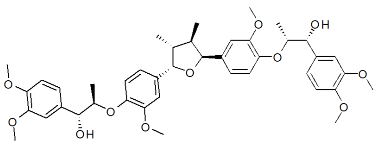 | *Saururuscernuus* L. (Saururaceae) | [20] |
| Piceatannol | 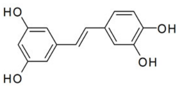 | Various fruits and vegetables such as grapes | [21] |

| | | | |
|---|---|---|---|
| Pterostilbene * (NCT03671811, endometrial cancer, recruiting) | 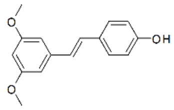 | Grapes and blueberries | [22] |
| Resveratrol | 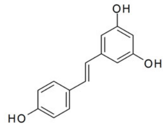 | Grapes and peanuts | [23] |
| Rhaponticin | 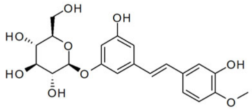 | Medicinal herbs such as *Rheum undulatum* L. | [24] |
| Vanillic acid | 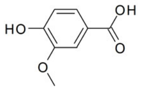 | *Angelica sinensis* and green tea | [25] |
| | **Other Polyphenols** | | |
| Chlorogenic acid | 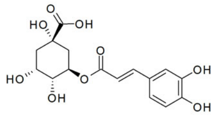 | Potatoes and apples | [26] |
| Curcumin * (NCT03980509, breast cancer, recruiting) (NCT01294072, colorectal cancer, recruiting) (NCT02064673, prostate cancer, recruiting) (NCT04403568, prostate cancer, recruiting) (NCT01740323, breast cancer, completed) | 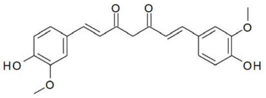 | The rhizome of *Curcuma longa* | [27] |
| Decursin | 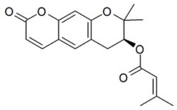 | The roots of *Angelica gigas* | [28] |
| DPHP | 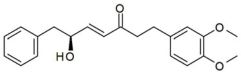 | An analogue of alpinoid c, which is from *Alpinia officinaram* | [29] |
| Gambogic acid | 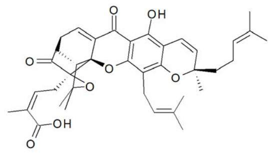 | *Garcinia hanburyi* | [30] |
| Garcinol | 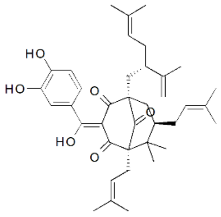 | The fruit rind of *Garcinia indica* | [31] |

| | | | |
|---|---|---|---|
| Imperatorin | 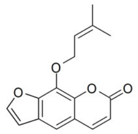 | *Angelica dahurica* | [32] |
| Salidroside | 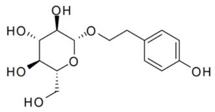 | *Rhodiola rosea* L. | [33] |
| Shikonin | 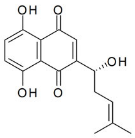 | The roots of *Lithospermum erythrorhizon* | [34] |
| Verbascoside | 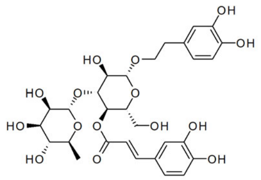 | Mullein (*Verbascum sinuatum* L.; Scrophulariaceae) | [35] |
| | **Terpenes** | | |
| β-elemene * (NCT02629757, anaplastic oligoastrocytoma/anaplastic astrocytoma/glioblastoma, recruiting) | 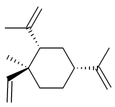 | *Curcuma wenyujin* | [36] |
| Andrographolide * (NCT04196075, esophageal cancer, recruiting) | 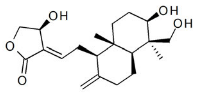 | *Andrographis paniculata* Nees (Acanthaceae) | [37] |
| Balanophorin B | 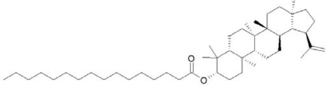 | *Balanophora spicata*, *B. indica*, and *B. simaoensis* | [38] |
| Betulinic acid | 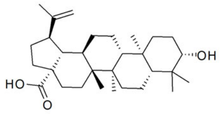 | Plants such as white-barked birch trees | [39] |
| Britannin | 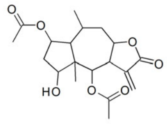 | *Inula Britannica* L. | [40] |
| Celastrol | 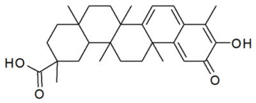 | *Tripterygium wilfordii* Hook F | [41] |
| Cephalomannine | 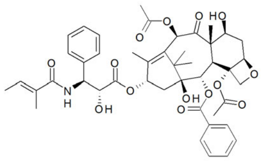 | *Taxus wallichiana* (yew species) | [42] |
| Cryptotanshinone | 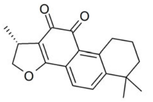 | *Salvia miltiorrhiza Bunge* | [43] |

| | | | |
|---|---|---|---|
| Curcumol | 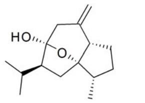 | *Curcuma wenyujin* | [44] |
| Ilexgenin A | 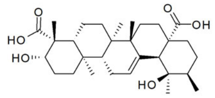 | *Ilex hainanensis* Merr | [45] |
| Kamebakaurin | 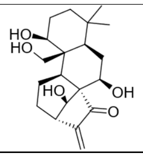 | *Isodon excia* (Maxim.) Hara | [46] |
| Micheliolide | 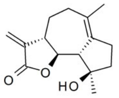 | *Michelia compressa* and *Michelia champaca* | [47] |
| Panaxadiol | 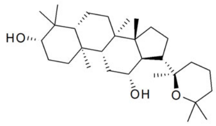 | The roots of *Panax ginseng* | [48] |
| Perillyl alcohol * (NCT02704858, glioblastoma multiforme, recruiting) (NCT00003219, breast cancer, completed) (NCT00003238, prostate cancer, completed) (NCT00003769, pancreatic cancer, completed) | 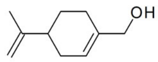 | Lavender, cherries, and mint | [49] |
| Pomolic acid | 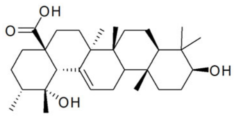 | *Euscaphis japonica* | [50] |
| Pristimerin | 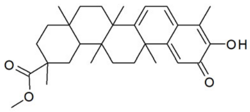 | Celastraceae and Hippocrateaceae families | [51] |
| Tanshinone IIA | 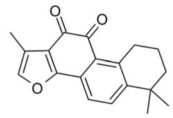 | The dried root of *Salvia miltiorrhiza* | [52] |
| Theasaponin E1 | 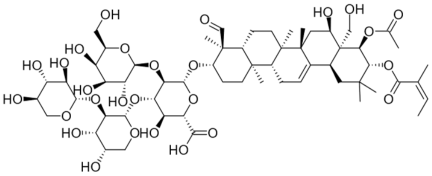 | *Camellia sinensis* seeds | [53] |
| Thymoquinone | 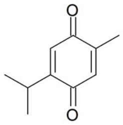 | *Nigella sativa* (black seed) | [54] |

| | | | |
|---|---|---|---|
| Triptolide * (NCT03129139, advanced solid cancers, recruiting) (NCT04896073, pancreatic cancer, recruiting) (NCT03117920, pancreatic cancer, completed) | 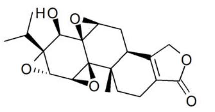 | *Tripterygium wilfordii* hook | [55] |
| Ursolic acid | 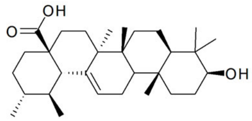 | A variety of natural plants, including *Hedyotic diffusa* Willd. and *Prunell avulgaris* L. | [56] |

* indicates compounds in clinical trials for cancer. The national clinical trial (NCT) number, condition/disease, and recruitment status registered in ClinicalTrials.gov are referred.

## Supplementary References


1. Pan, Y.; Shao, D.; Zhao, Y.; Zhang, F.; Zheng, X.; Tan, Y.; He, K.; Li, J.; Chen, L. Berberine reverses hypoxia-induced chemoresistance in breast cancer through the inhibition of ampk- hif-1alpha. *Int J Biol Sci* **2017**, *13*, 794-803.
2. Kalainayakan, S.P.; Ghosh, P.; Dey, S.; Fitzgerald, K.E.; Sohoni, S.; Konduri, P.C.; Garrossian, M.; Liu, L.; Zhang, L. Cyclopamine tartrate, a modulator of hedgehog signaling and mitochondrial respiration, effectively arrests lung tumor growth and progression. *Sci Rep* **2019**, *9*, 1405.
3. Du, Y.; Zheng, Z.G.; Yu, Y.; Wu, Z.T.; Liang, D.; Li, P.; Jiang, Y.; Li, H.J. Rapid discovery of cyclopamine analogs from fritillaria and veratrum plants using lc-q-tof-ms and lc-qqq-ms. *J Pharm Biomed Anal* **2017**, *142*, 201-209.
4. Wang, J.Y.; Wang, Z.; Li, M.Y.; Zhang, Z.; Mi, C.; Zuo, H.X.; Xing, Y.; Wu, Y.L.; Lian, L.H.; Xu, G.H., *et al.* Dictamnine promotes apoptosis and inhibits epithelial-mesenchymal transition, migration, invasion and proliferation by downregulating the hif-1alpha and slug signaling pathways. *Chem Biol Interact* **2018**, *296*, 134-144.
5. Liu, R.M.; Xu, P.; Chen, Q.; Feng, S.L.; Xie, Y. A multiple-targets alkaloid nuciferine overcomes paclitaxel-induced drug resistance in vitro and in vivo. *Phytomedicine* **2020**, *79*, 153342.
6. Su, Q.; Wang, J.; Wu, Q.; Ullah, A.; Ghauri, M.A.; Sarwar, A.; Chen, L.; Liu, F.; Zhang, Y. Sanguinarine combats hypoxia-induced activation of ephb4 and hif-1alpha pathways in breast cancer. *Phytomedicine* **2021**, *84*, 153503.
7. Chen, Z.; Zhao, L.; Zhao, F.; Yang, G.; Wang, J.J. Tetrandrine suppresses lung cancer growth and induces apoptosis, potentially via the vegf/hif-1alpha/icam-1 signaling pathway. *Oncol Lett* **2018**, *15*, 7433-7437.
8. Wei, Z.; Shan, Y.; Tao, L.; Liu, Y.; Zhu, Z.; Liu, Z.; Wu, Y.; Chen, W.; Wang, A.; Lu, Y. Diallyl trisulfides, a natural histone deacetylase inhibitor, attenuate hif-1alpha synthesis, and decreases breast cancer metastasis. *Mol Carcinog* **2017**, *56*, 2317-2331.
9. Xia, Y.; Kang, T.W.; Jung, Y.D.; Zhang, C.; Lian, S. Sulforaphane inhibits nonmuscle invasive bladder cancer cells proliferation through suppression of hif-1alpha-mediated glycolysis in hypoxia. *J Agric Food Chem* **2019**, *67*, 7844-7854.
10. Shankar, E.; Goel, A.; Gupta, K.; Gupta, S. Plant flavone apigenin: An emerging anticancer agent. *Curr Pharmacol Rep* **2017**, *3*, 423-446.
11. Jin, J.; Qiu, S.; Wang, P.; Liang, X.; Huang, F.; Wu, H.; Zhang, B.; Zhang, W.; Tian, X.; Xu, R., *et al.* Cardamonin inhibits breast cancer growth by repressing hif-1alpha-dependent metabolic reprogramming. *J Exp Clin Cancer Res* **2019**, *38*, 377.
12. Luo, H.Q.; Xu, M.; Zhong, W.T.; Cui, Z.Y.; Liu, F.M.; Zhou, K.Y.; Li, X.Y. Egcg decreases the expression of hif-1alpha and vegf and cell growth in mcf-7 breast cancer cells. *J BUON* **2014**, *19*, 435-439.
13. Guo, Q.; Lu, L.; Liao, Y.; Wang, X.; Zhang, Y.; Liu, Y.; Huang, S.; Sun, H.; Li, Z.; Zhao, L. Influence of c-src on hypoxic resistance to paclitaxel in human ovarian cancer cells and reversal of fv-429. *Cell Death Dis* **2018**, *8*, e3178.

14. Wei, M.; Ma, R.; Huang, S.; Liao, Y.; Ding, Y.; Li, Z.; Guo, Q.; Tan, R.; Zhang, L.; Zhao, L. Oroxylin a increases the sensitivity of temozolomide on glioma cells by hypoxia-inducible factor 1alpha/hedgehog pathway under hypoxia. *J Cell Physiol* **2019**, *234*, 17392-17404.
15. Wang, S.J.; Zhao, J.K.; Ren, S.; Sun, W.W.; Zhang, W.J.; Zhang, J.N. Wogonin affects proliferation and the energy metabolism of sgc-7901 and a549 cells. *Exp Ther Med* **2019**, *17*, 911-918.
16. Wen, Y.; Zhao, R.Q.; Zhang, Y.K.; Gupta, P.; Fu, L.X.; Tang, A.Z.; Liu, B.M.; Chen, Z.S.; Yang, D.H.; Liang, G. Effect of y6, an epigallocatechin gallate derivative, on reversing doxorubicin drug resistance in human hepatocellular carcinoma cells. *Oncotarget* **2017**, *8*, 29760-29770.
17. Kim, D.H.; Sung, B.; Kim, J.A.; Kang, Y.J.; Hwang, S.Y.; Hwang, N.L.; Suh, H.; Choi, Y.H.; Im, E.; Chung, H.Y., *et al.* Hs-1793, a resveratrol analogue, downregulates the expression of hypoxia-induced hif-1 and vegf and inhibits tumor growth of human breast cancer cells in a nude mouse xenograft model. *Int J Oncol* **2017**, *51*, 715-723.
18. Lai, F.; Liu, Q.; Liu, X.; Ji, M.; Xie, P.; Chen, X. Lxy6090 - a novel manassantin a derivative - limits breast cancer growth through hypoxia-inducible factor-1 inhibition. *Onco Targets Ther* **2016**, *9*, 3829-3840.
19. Chen, M.C.; Lee, C.F.; Huang, W.H.; Chou, T.C. Magnolol suppresses hypoxia-induced angiogenesis via inhibition of hif-1alpha/vegf signaling pathway in human bladder cancer cells. *Biochem Pharmacol* **2013**, *85*, 1278-1287.
20. Kwak, S.H.; Stephenson, T.N.; Lee, H.E.; Ge, Y.; Lee, H.; Min, S.M.; Kim, J.H.; Kwon, D.Y.; Lee, Y.M.; Hong, J. Evaluation of manassantin a tetrahydrofuran core region analogues and cooperative therapeutic effects with egfr inhibition. *J Med Chem* **2020**, *63*, 6821-6833.
21. Aljabali, A.A.A.; Bakshi, H.A.; Hakkim, F.L.; Haggag, Y.A.; Al-Batanyeh, K.M.; Al Zoubi, M.S.; Al-Trad, B.; Nasef, M.M.; Satija, S.; Mehta, M., *et al.* Albumin nano-encapsulation of piceatannol enhances its anticancer potential in colon cancer via downregulation of nuclear p65 and hif-1alpha. *Cancers (Basel)* **2020**, *12*.
22. Butt, N.A.; Kumar, A.; Dhar, S.; Rimando, A.M.; Akhtar, I.; Hancock, J.C.; Lage, J.M.; Pound, C.R.; Lewin, J.R.; Gomez, C.R., *et al.* Targeting mta1/hif-1alpha signaling by pterostilbene in combination with histone deacetylase inhibitor attenuates prostate cancer progression. *Cancer Med* **2017**, *6*, 2673-2685.
23. Wang, H.; Jia, R.; Lv, T.; Wang, M.; He, S.; Zhang, X. Resveratrol suppresses tumor progression via inhibiting stat3/hif-1alpha/vegf pathway in an orthotopic rat model of non-small-cell lung cancer (nsclc). *Onco Targets Ther* **2020**, *13*, 7057-7063.
24. Kim, A.; Ma, J.Y. Rhaponticin decreases the metastatic and angiogenic abilities of cancer cells via suppression of the hif1alpha pathway. *Int J Oncol* **2018**, *53*, 1160-1170.
25. Gong, J.; Zhou, S.; Yang, S. Vanillic acid suppresses hif-1alpha expression via inhibition of mtor/p70s6k/4e-bp1 and raf/mek/erk pathways in human colon cancer hct116 cells. *Int J Mol Sci* **2019**, *20*.
26. Park, J.J.; Hwang, S.J.; Park, J.H.; Lee, H.J. Chlorogenic acid inhibits hypoxia-induced angiogenesis via down-regulation of the hif-1alpha/akt pathway. *Cell Oncol (Dordr)* **2015**, *38*, 111-118.
27. Monteleone, F.; Taverna, S.; Alessandro, R.; Fontana, S. Swath-ms based quantitative proteomics analysis reveals that curcumin alters the metabolic enzyme profile of cml cells by affecting the activity of mir-22/ipo7/hif-1alpha axis. *J Exp Clin Cancer Res* **2018**, *37*, 170.
28. Ge, Y.; Yoon, S.H.; Jang, H.; Jeong, J.H.; Lee, Y.M. Decursin promotes hif-1alpha proteasomal degradation and immune responses in hypoxic tumour microenvironment. *Phytomedicine* **2020**, *78*, 153318.
29. Velatooru, L.R.; Vakamullu, S.; Penugurti, V.; S, P.R. Alpinoid c analog inhibits angiogenesis and induces apoptosis in colo205cell line. *Chem Biol Interact* **2019**, *308*, 1-10.
30. Wang, F.; Zhang, W.; Guo, L.; Bao, W.; Jin, N.; Liu, R.; Liu, P.; Wang, Y.; Guo, Q.; Chen, B. Gambogic acid suppresses hypoxia-induced hypoxia-inducible factor-1alpha/vascular endothelial growth factor expression via inhibiting phosphatidylinositol 3-kinase/akt/mammalian target protein of rapamycin pathway in multiple myeloma cells. *Cancer Sci* **2014**, *105*, 1063-1070.
31. Ranjbarnejad, T.; Saidijam, M.; Tafakh, M.S.; Pourjafar, M.; Talebzadeh, F.; Najafi, R. Garcinol exhibits anti-proliferative activities by targeting microsomal prostaglandin e synthase-1 in human colon cancer cells. *Hum Exp Toxicol* **2017**, *36*, 692-700.
32. Mi, C.; Ma, J.; Wang, K.S.; Zuo, H.X.; Wang, Z.; Li, M.Y.; Piao, L.X.; Xu, G.H.; Li, X.; Quan, Z.S., *et al.* Imperatorin suppresses proliferation and angiogenesis of human colon cancer cell by targeting hif-1alpha via the mtor/p70s6k/4e-bp1 and mapk pathways. *J Ethnopharmacol* **2017**, *203*, 27-38.

33. Qin, Y.; Liu, H.J.; Li, M.; Zhai, D.H.; Tang, Y.H.; Yang, L.; Qiao, K.L.; Yang, J.H.; Zhong, W.L.; Zhang, Q., *et al.* Salidroside improves the hypoxic tumor microenvironment and reverses the drug resistance of platinum drugs via hif-1alpha signaling pathway. *EBioMedicine* **2018**, *38*, 25-36.
34. Li, M.Y.; Mi, C.; Wang, K.S.; Wang, Z.; Zuo, H.X.; Piao, L.X.; Xu, G.H.; Li, X.; Ma, J.; Jin, X. Shikonin suppresses proliferation and induces cell cycle arrest through the inhibition of hypoxia-inducible factor-1alpha signaling. *Chem Biol Interact* **2017**, *274*, 58-67.
35. Attia, Y.M.; El-Kersh, D.M.; Wagdy, H.A.; Elmazar, M.M. Verbascoside: Identification, quantification, and potential sensitization of colorectal cancer cells to 5-fu by targeting pi3k/akt pathway. *Sci Rep* **2018**, *8*, 16939.
36. Yu, X.; Li, Z.; Zhang, Y.; Xu, M.; Che, Y.; Tian, X.; Wang, R.; Zou, K.; Zou, L. Beta-elemene inhibits radiation and hypoxia-induced macrophages infiltration via prx-1/nf-kappab/hif-1alpha signaling pathway. *Onco Targets Ther* **2019**, *12*, 4203-4211.
37. Shi, L.; Zhang, G.; Zheng, Z.; Lu, B.; Ji, L. Andrographolide reduced vegfa expression in hepatoma cancer cells by inactivating hif-1alpha: The involvement of jnk and mta1/hdca. *Chem Biol Interact* **2017**, *273*, 228-236.
38. Dai, T.; Li, L.; Qi, W.; Liu, B.; Jiang, Z.; Song, J.; Hua, H. Balanophorin b inhibited glycolysis with the involvement of hif-1alpha. *Life Sci* **2021**, *267*, 118910.
39. Kim, H.J.; Cho, H.S.; Ban, H.S.; Nakamura, H. Suppression of hif-1alpha accumulation by betulinic acid through proteasome activation in hypoxic cervical cancer. *Biochem Biophys Res Commun* **2020**, *523*, 726-732.
40. Zhang, Y.F.; Zhang, Z.H.; Li, M.Y.; Wang, J.Y.; Xing, Y.; Ri, M.; Jin, C.H.; Xu, G.H.; Piao, L.X.; Zuo, H.X., *et al.* Britannin stabilizes t cell activity and inhibits proliferation and angiogenesis by targeting pd-l1 via abrogation of the crosstalk between myc and hif-1alpha in cancer. *Phytomedicine* **2021**, *81*, 153425.
41. Zhu, Y.; Liu, X.; Zhao, P.; Zhao, H.; Gao, W.; Wang, L. Celastrol suppresses glioma vasculogenic mimicry formation and angiogenesis by blocking the pi3k/akt/mtor signaling pathway. *Front Pharmacol* **2020**, *11*, 25.
42. Ullah, A.; Leong, S.W.; Wang, J.; Wu, Q.; Ghauri, M.A.; Sarwar, A.; Su, Q.; Zhang, Y. Cephalomannine inhibits hypoxia-induced cellular function via the suppression of apex1/hif-1alpha interaction in lung cancer. *Cell Death Dis* **2021**, *12*, 490.
43. Zhang, L.; Chen, C.; Duanmu, J.; Wu, Y.; Tao, J.; Yang, A.; Yin, X.; Xiong, B.; Gu, J.; Li, C., *et al.* Cryptotanshinone inhibits the growth and invasion of colon cancer by suppressing inflammation and tumor angiogenesis through modulating mmp/timp system, pi3k/akt/mtor signaling and hif-1alpha nuclear translocation. *Int Immunopharmacol* **2018**, *65*, 429-437.
44. Zuo, H.X.; Jin, Y.; Wang, Z.; Li, M.Y.; Zhang, Z.H.; Wang, J.Y.; Xing, Y.; Ri, M.H.; Jin, C.H.; Xu, G.H., *et al.* Curcumol inhibits the expression of programmed cell death-ligand 1 through crosstalk between hypoxia-inducible factor-1alpha and stat3 (t705) signaling pathways in hepatic cancer. *J Ethnopharmacol* **2020**, *257*, 112835.
45. Zhang, L.; Qiao, X.; Chen, M.; Li, P.; Wen, X.; Sun, M.; Ma, X.; Hou, Y.; Yang, J. Ilexgenin a prevents early colonic carcinogenesis and reprogramed lipid metabolism through hif1alpha/srebp-1. *Phytomedicine* **2019**, *63*, 153011.
46. Wang, K.S.; Ma, J.; Mi, C.; Li, J.; Lee, J.J.; Jin, X. Kamebakaurin inhibits the expression of hypoxia-inducible factor-1alpha and its target genes to confer antitumor activity. *Oncol Rep* **2016**, *35*, 2045-2052.
47. Kong, P.; Yu, K.N.; Yang, M.; Almahi, W.A.; Nie, L.; Chen, G.; Han, W. Micheliolide enhances radiosensitivities of p53-deficient non-small-cell lung cancer via promoting hif-1alpha degradation. *Int J Mol Sci* **2020**, *21*.
48. Wang, Z.; Li, M.Y.; Zhang, Z.H.; Zuo, H.X.; Wang, J.Y.; Xing, Y.; Ri, M.; Jin, H.L.; Jin, C.H.; Xu, G.H., *et al.* Panaxadiol inhibits programmed cell death-ligand 1 expression and tumour proliferation via hypoxia-inducible factor (hif)-1alpha and stat3 in human colon cancer cells. *Pharmacol Res* **2020**, *155*, 104727.
49. Ma, J.; Li, J.; Wang, K.S.; Mi, C.; Piao, L.X.; Xu, G.H.; Li, X.; Lee, J.J.; Jin, X. Perillyl alcohol efficiently scavenges activity of cellular ros and inhibits the translational expression of hypoxia-inducible factor-1alpha via mtor/4e-bp1 signaling pathways. *Int Immunopharmacol* **2016**, *39*, 1-9.
50. Park, J.H.; Yoon, J.; Park, B. Pomolic acid suppresses hif1alpha/vegf-mediated angiogenesis by targeting p38-mapk and mtor signaling cascades. *Phytomedicine* **2016**, *23*, 1716-1726.
51. Costa, P.M.; Ferreira, P.M.; Bolzani Vda, S.; Furlan, M.; de Freitas Formenton Macedo Dos Santos, V.A.; Corsino, J.; de Moraes, M.O.; Costa-Lotufo, L.V.; Montenegro, R.C.; Pessoa, C. Antiproliferative activity of pristimerin isolated from maytenus ilicifolia (celastraceae) in human hl-60 cells. *Toxicol In Vitro* **2008**, *22*, 854-863.
52. Li, G.; Shan, C.; Liu, L.; Zhou, T.; Zhou, J.; Hu, X.; Chen, Y.; Cui, H.; Gao, N. Tanshinone iia inhibits hif-1alpha and vegf expression in breast cancer cells via mtor/p70s6k/rps6/4e-bp1 signaling pathway. *PLoS One* **2015**, *10*, e0117440.

53. Li, B.; Tong, T.; Ren, N.; Rankin, G.O.; Rojanasakul, Y.; Tu, Y.; Chen, Y.C. Theasaponin e1 inhibits platinum-resistant ovarian cancer cells through activating apoptosis and suppressing angiogenesis. *Molecules* **2021**, *26*.
54. Lee, Y.M.; Kim, G.H.; Park, E.J.; Oh, T.I.; Lee, S.; Kan, S.Y.; Kang, H.; Kim, B.M.; Kim, J.H.; Lim, J.H. Thymoquinone selectively kills hypoxic renal cancer cells by suppressing hif-1alpha-mediated glycolysis. *Int J Mol Sci* **2019**, *20*.
55. Ding, X.; Zhou, X.; Jiang, B.; Zhao, Q.; Zhou, G. Triptolide suppresses proliferation, hypoxia-inducible factor-1alpha and c-myc expression in pancreatic cancer cells. *Mol Med Rep* **2015**, *12*, 4508-4513.
56. Shan, J.Z.; Xuan, Y.Y.; Zhang, Q.; Huang, J.J. Ursolic acid sensitized colon cancer cells to chemotherapy under hypoxia by inhibiting mdr1 through hif-1alpha. *J Zhejiang Univ Sci B* **2016**, *17*, 672-682.
